# Pedagogical Merit Review of Animal Use for Education in Canada

**DOI:** 10.1371/journal.pone.0158002

**Published:** 2016-06-28

**Authors:** Marc T. Avey, Gilly Griffin

**Affiliations:** 1 Faculty of Medicine, University of Ottawa, Ottawa, Ontario, Canada; 2 Clinical Epidemiology Program, Ottawa Hospital Research Institute, Ottawa, Ontario, Canada; 3 Canadian Council on Animal Care, Ottawa, Ontario, Canada; Universidade do Porto Instituto de Biologia Molecular e Celular, PORTUGAL

## Abstract

There are two components to the review of animal based protocols in Canada: review for the merit of the study itself, and review of the ethical acceptability of the work. Despite the perceived importance for the quality assurance these reviews provide; there are few studies of the peer-based merit review system for animal-based protocols for research and education. Institutional animal care committees (ACC)s generally rely on the external peer review of scientific merit for animal-based research. In contrast, peer review for animal based teaching/training is dependent on the review of pedagogical merit carried out by the ACC itself or another committee within the institution. The objective of this study was to evaluate the views of ACC members about current practices and policies as well as alternate policies for the review of animal based teaching/training. We conducted a national web-based survey of ACC members with both quantitative and qualitative response options. Responses from 167 ACC members indicated broad concerns about administrative burden despite strong support for both the current and alternate policies. Participants’ comments focused mostly on the merit review process (54%) relative to the efficiency (21%), impact (13%), and other (12%) aspects of evaluation. Approximately half (49%) of the comments were classified into emergent themes that focused on some type of burden: burden from additional pedagogical merit review (16%), a limited need for the review (12%), and a lack of resources (expertise 11%; people/money 10%). Participants indicated that the current system for pedagogical merit review is effective (60%); but most also indicated that there was at least some challenge (86%) with the current peer review process. There was broad support for additional guidance on the justification, criteria, types of animal use, and objectives of pedagogical merit review. Participants also supported the ethical review and application of the Three Rs in the review process. A clear priority from participants in the survey was updating guidance to better facilitate the merit review process of animal-based protocols for education. Balancing the need for improved guidance with the reality of limited resources at local institutions will be essential to do this successfully; a familiar dilemma to both scientists and policy makers alike.

## Introduction

Peer review is considered to be the cornerstone of sound science [1]. The peer review of animal-based studies, is similarly considered to be a cornerstone of the system overseeing animal-based science. There are two components to the review of animal based protocols: the review for merit of the study itself, and the review of the ethical acceptability of the work. In Canada, institutional animal care committees (ACCs) generally rely on the external review of scientific merit for animal-based research [2,3]; although every institution must have an internal mechanism to provide scientific peer review if necessary. In contrast, review for animal based teaching/training is dependent on the review of pedagogical merit carried out by the ACC itself or another committee within the institution [4–8].

The Canadian Council on Animal Care (CCAC) is the organization which provides nationally and internationally-recognized standards for the ethics and care of animals in science and verifies their effective implementation in Canadian institutions. Peer review by the ACC for the merit of animal protocols is a CCAC requirement, as outlined in its guidelines and policies [9]. The CCAC has evolved its guidance on pedagogical merit review over time. Currently there are two guidelines and three policy documents which pertain to animal-based teaching/training [4–8]. The term ‘pedagogical’ was first introduced in the *Guide to the Care and Use of Experimental Animals* [5] as well as the idea of a separate committee establishing the ‘pedagogical merit’ for animal-based courses, which would then form part of the protocol submission to the ACC. This concept was updated and expanded in two subsequent policy documents [6,8] which further outlined the responsibilities of the local ACCs. This left the ACC at liberty to conduct a pedagogical merit review itself or to call upon an institutional curriculum committee to provide the review [5]. The 2006 policy update [8] directed ACCs to the *CCAC guidelines on*: *animal use protocol review* (1997) which provided guidance on what should be evaluated during the review. These guidelines and CCAC’s overarching policy frame animal-based teaching/training as ‘markedly different’ than animal-based research and recommend items that reviewers may consider during the review such as justification for animal-based teaching/training over the use of non-animal alternatives.

**Fig 1:**
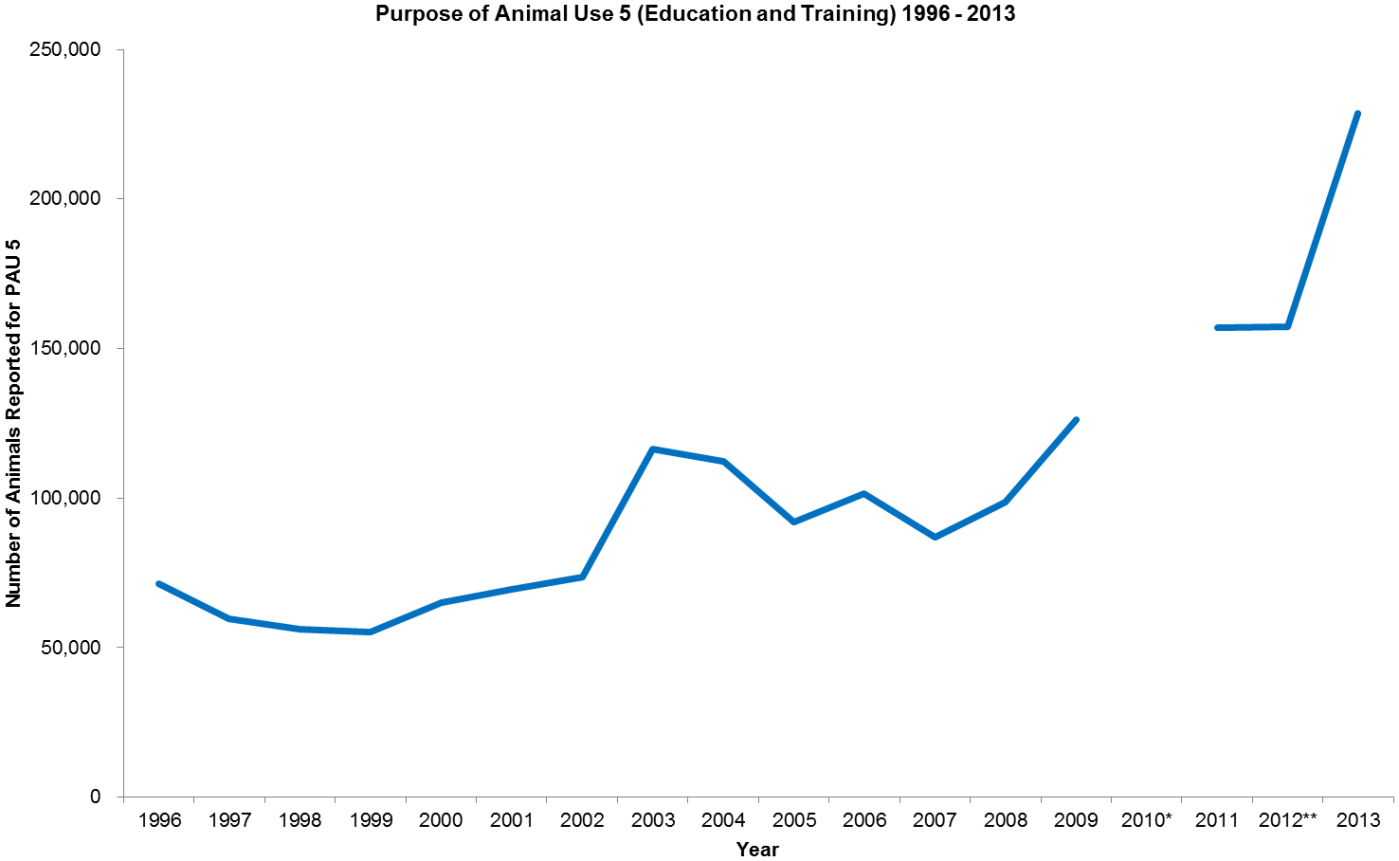
Purpose of Animal Use 5 (Education and Training) 1996–2013. *Data from 2010 was unavailable. ** Data from 2012 onwards was collected by including re-use of animals; whereas animal use prior to 2012 only included single animal use based on the highest category of invasiveness. Thus, 2012 animal use numbers may be higher if animals are used more than once as this information is now captured. See http://www.ccac.ca/Documents/AUD/2012-Animal-Data-Report.pdf for more information.

It is also a CCAC requirement that the Three Rs (replacement, reduction, refinement) be considered and applied by ACCs during peer review for ethics of animal-based protocols. Replacement is emphasized for—animal-based teaching/training such that animal-based studies are approved “…only if the researcher's best efforts to find an alternative have failed” [4]. Despite this restriction, the percentage of animals for animal-based teaching/training has remained stable between three and five percent of the total number animals reported since 1996. From 1996 to 2013, the total number of animals reported for teaching/training (Purpose of Animal Use 5 [10]) was 1,726,684 (mean 101,570; median 91,879; range 55,267–228,759; Fig 1). However, the total number of animals used may be currently under-reported as the purchase of dead animals from biological supply houses is not reported to the CCAC. Given the absolute increase in the number of animals used, the merit review process and policies around animal-based teaching/training should be formally assessed since they have not been evaluated since the *Ethics of Animal Investigation* was published in 1989 [4].

The objective of this study was to evaluate 1) the current practices in place at institutions to conduct pedagogical merit of animal use protocols; 2) how current CCAC guidance/policy on merit review is viewed by participants; and 3) how alternate guidance/policy recommendations are viewed.

## Methods

### Reporting and Ethics

We followed the reporting standards for surveys [11,12] as described by the EQUATOR Network. Ethics approval and an extension were granted from Institutional Review Board Services (http://www.irbservices.com). The protocol, and extension numbers were: Final Protocol Number and Date: Feasibility Study Version date March 28th, 2012; and Final Protocol Number and Date: Feasibility Study Version date March 28th, 2012. Modifications and French translations were approved April 19^th^ 2013. Informed consent was obtained for each participant in the interview portion of the survey by having them read and sign an information and consent form prior to conducting the interview. Informed consent was obtained for each participant in the on-line survey by having them read and electronically agree to participate as the first step in conducting the survey. None of the 12 individuals contacted for the interview survey declined to participate, and only one of the 266 participants who completed the informed consent for the on-line survey declined. There was no apparent difference between the participant who declined to participate and the remainder of the cohort.

### Survey Development

In light of informal feedback from ACCs concerning the lack of formal guidance or policy to assist with the pedagogical merit review of animal teaching/training protocols, a CCAC expert working group on pedagogical merit was established in 2010. The working group drafted a set of recommendations. Based on these recommendations, the then CCAC Guidelines committee requested that an evaluation of current and proposed changes to the peer review policy around pedagogical merit review be conducted.

Individual interviews were conducted via phone with key stakeholders from institutions to assist in the development of the on-line survey of CCAC stakeholders. A question guide for these individual interviews was developed with content based on current CCAC guidance, and new guidance recommendations from the CCAC expert working group. The content validity [13], was assessed by members of the CCAC Secretariat, experts in animal based teaching/training, and individuals with experience reviewing animal protocols for teaching and training.

For the interviews, the sample was purposive with the aim of selecting individuals that had experience with the use of animals in teaching/training from across Canada. We contacted all ACC coordinators from institutions which participate in the CCAC’s program as well as a senior administrator from each of these institutions. In addition we also contacted national organizations (e.g. Canadian Association of Laboratory Animal Science; Canadian Veterinary Medical Association); and student associations (both graduate and undergraduate). We interviewed twelve individuals from academic institutions who are involved with the animal based teaching/training and stopped interviewing when theoretical saturation was reached [14,15].

Feedback from the individual interviews was combined with the expert working group recommendations to develop a set of core questions for the survey. Efforts were made to include as many of the issues raised by both the interviewees and the working group as possible; however, for practical reasons the survey had to be limited in its scope and number of questions. Although both the interviewees and the working group members preferred the term ‘educational’ over ‘pedagogical’, for the on-line survey we used ‘pedagogical’ for consistency with official terminology in CCAC guidelines and policy statements. To assess the content validity, the on-line survey was reviewed by members of the CCAC Secretariat as well as external experts in education and evaluation.

The survey was developed in English and translated to French by a professional translator and then the translation was certified by the IRB. The survey was administered online using SurveyMonkey (http://www.surveymonkey.com). Piloting of the survey for functionality and usability was conducted by MTA and GG. There were 22 questions (including the information and consent decision), of which 19 required responses to proceed, and 10 questions had optional comment boxes (S1 Appendix). The survey was ten pages in length (two pages for the information and consent with decision question on the second page), with a median two questions per page (range = 1–6). Participants were unable to review their responses after they completed the final question. The survey was made open, such that anyone provided with a link could participate. The first item was for the informed consent decision. Items 2–7 were demographics related questions, items 8–21 were the opinion survey questions, and item 22 was a question aimed at soliciting general feedback to the CCAC. For the survey questions 8–20 a response on a five-point Likert scale (Strongly Agree, Agree, Neutral, Disagree, Strongly Disagree) was required [16]. Eight of the five-point Likert items also provided space for optional comments. The final two questions were optional free-form responses and asked what the most significant challenge for pedagogical merit review (question 21), and for any other comments related to the CCAC in general (question 22). Question 22 was not analyzed for this paper.

### Survey Sampling Frame

The sampling frame for the survey was all institutions that participate in the CCAC Program. Initially potential organizations/individuals were selected in a purposive manner based on their affiliation with academic, government, and private Canadian institutions that participate in the CCAC program. We contacted ACC coordinators, senior administrators, individuals responsible for providing institutional animal data, relevant national organizations, CCAC member representatives, and undergraduate and graduate student association contacts. We used a snowball sampling method [17] where we asked organizations/individuals we contacted to forward the email to any relevant individuals. In addition to the email solicitation, the CCAC advertised the survey on their website. We sent the initial email solicitation on June 17^th^ 2013, a reminder email on July 8^th^ 2013, and we closed the survey on July 29^th^ 2013. The recruitment email message was sent to 611 organizations/individuals with the request that potential participants forward the request “to any individuals or groups who are involved in education using animals (e.g. administrators, instructors, students) that you believe would be interested in participating.” Only fully completed surveys were included in the analysis. No incentives were offered to participants for completing the survey.

### Survey Analysis

For the analysis of the 13 five-point Likert scale questions, responses for “Strongly Agree” and “Agree” were aggregated, as well as “Strongly Disagree” and “Disagree”[18]. We assessed whether participant experience as an instructor for animal-based teaching/training or conducting pedagogical merit review of animal-based teaching/training influenced the responses to questions 8 and 10.

An organizing framework was developed for the qualitative synthesis of the comments. All individual comments were coded into three schemes: 1) Agreement/Disagreement; 2) Aspect of Evaluation; and 3) Emergent Themes. For Agreement/Disagreement responses were coded according to whether they agreed with the statement, disagreed, or were neutral. For Aspect of Evaluation the responses were coded as into four categories: efficiency, impact, process, other [19]. For Emergent Themes the responses were coded based on any emergent themes that were identified and representative quotations are presented.

## Results

### Current Practice at Institutions (Question 8, 21)

**Fig 2:**
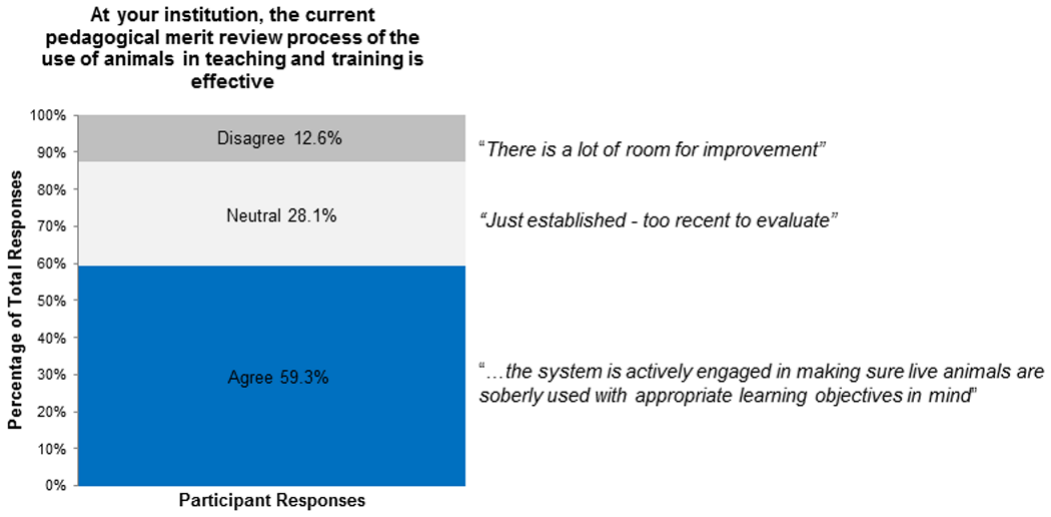
At your institution, the current pedagogical merit review process of the use of animals in teaching and training is effective.

**Fig 3:**
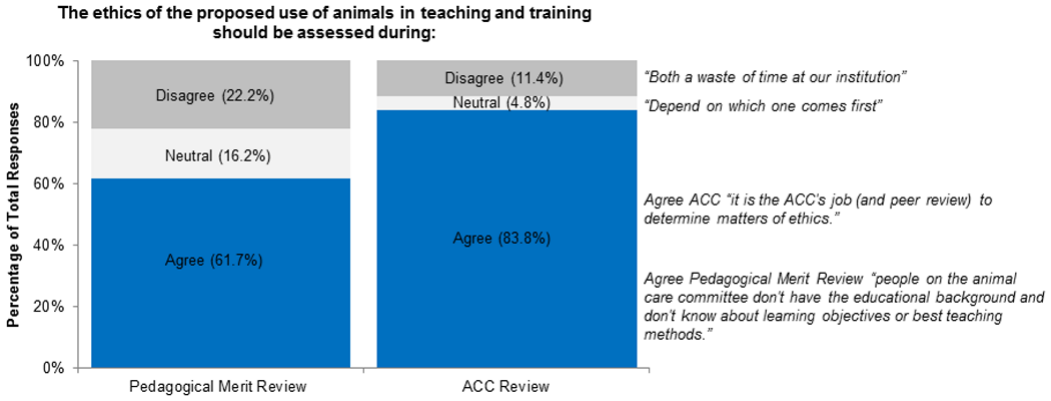
The ethics of the proposed use of animals in teaching and training should be assessed during.

**Fig 4:**
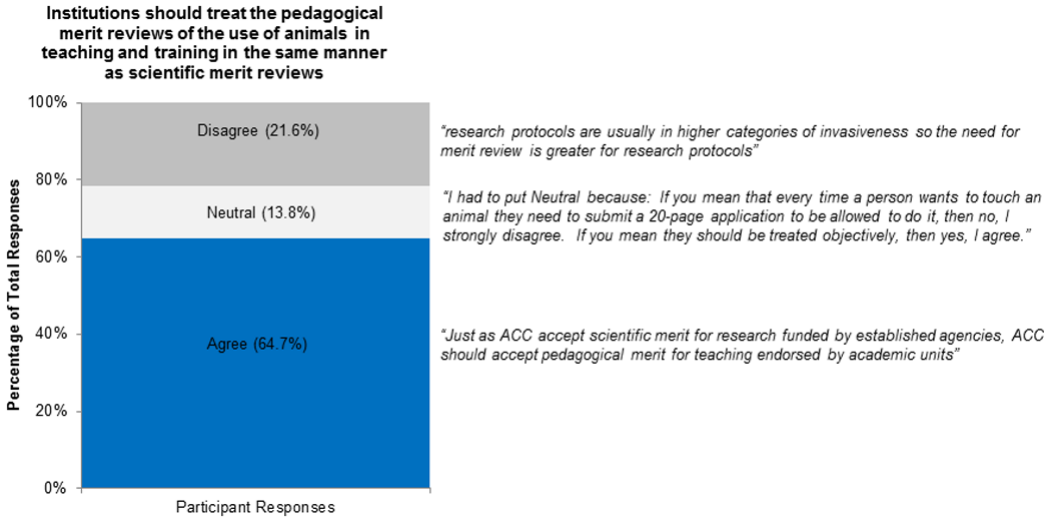
Institutions should treat the pedagogical merit reviews of the use of animals in teaching and training in the same manner as scientific merit reviews.

**Fig 5:**
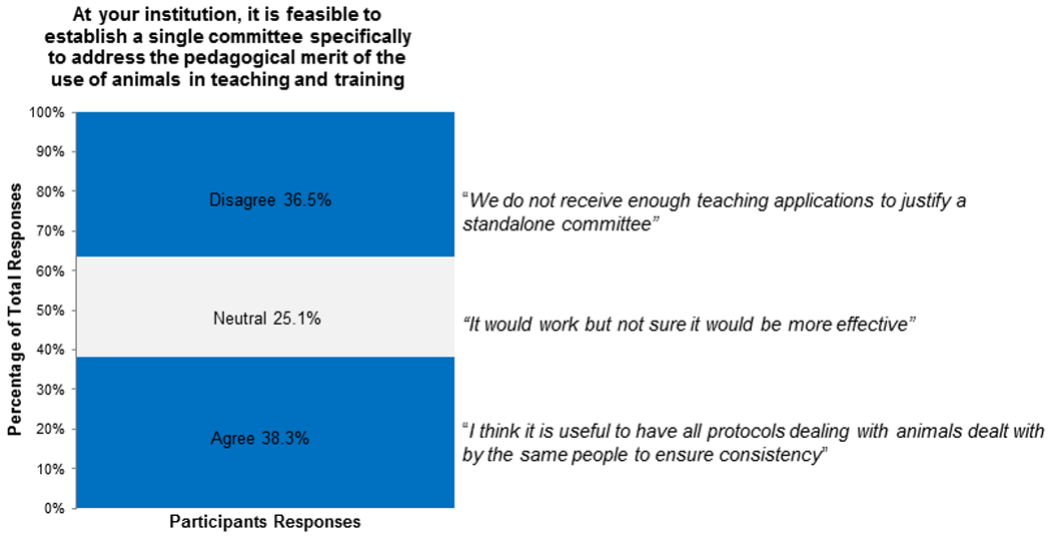
At your institution, it is feasible to establish a single committee specifically to address the pedagogical merit of the use of animals in teaching and training.

For Figs 2–5 we have included representative quotes in the figures to illustrate participants’ agreement/neutral/disagreement to the questions in the remaining sections. Descriptive statistics for questions 8–20 (question 21 was free form) are available in Table 1.

**Table 1:** Quantitative Responses for Questions 8–20

#	Question	Agree	Neutral	Disagree
		% (n)	% (n)	% (n)
8	At your institution, the current pedagogical merit review process of the use of animals in teaching and training is effective	59.3 (99)	28.1 (47)	12.6 (21)
9	The pedagogical merit review of the use of animals in teaching and training should be conducted by:			
9a	A committee(s) that is separate from the Animal Care Committee	45.5 (76)	18.0 (30)	35.9 (60)
9b	A curriculum committee(s)	42.5 (71)	22.8 (38)	34.1 (57)
9c	A single committee dedicated to the pedagogical merit review of the use of animals	43.7 (73)	19.8 (33)	35.3 (59)
9d	A national peer review committee	16.8 (28)	18.0 (30)	64.7 (108)
10	At your institution, it is feasible to establish a single committee specifically to address the pedagogical merit of the use of animals in teaching and training	38.3 (64)	25.1 (42)	36.5 (61)
11	A pedagogical merit review of the use of animals in teaching and training should be conducted by a group that includes:			
11a	Community members	58.1 (97)	16.8 (28)	25.1 (42)
11b	Experts in education	76.6 (128)	15.0 (25)	8.4 (14)
11c	Instructors who use animals in teaching/training	92.2 (154)	4.2 (7)	3.6 (6)
11d	Students (Undergraduate)	52.1 (87)	24.6 (41)	23.4 (39)
11e	Students (Graduate)	61.1 (102)	26.9 (45)	12.0 (20)
11f	Veterinarians	83.8 (140)	9.0 (15)	7.2 (12)
12	Institutions should treat the pedagogical merit reviews of the use of animals in teaching and training in the same manner as scientific merit reviews	64.7 (108)	13.8 (23)	21.6 (36)
13	The pedagogical merit review of the use of animals in teaching and training should evaluate:			
13a	The overall pedagogical merit of the course that the animal use takes place in	77.8 (130)	6.0 (10)	16.2 (27)
13b	If the use of animals is essential for meeting the education objectives of the learning session	92.8 (155)	4.2 (7)	3.0 (5)
13c	If the use of animals is essential for meeting the education objectives of the Course	94.6 (158)	3.6 (6)	1.8 (3)
13d	If the use of animals is essential for meeting the education objectives of the program	88.0 (147)	6.6 (11)	5.4 (9)
14	The pedagogical merit of the use of animals in teaching and training should determine whether:			
14a	The use of non-animal alternatives could meet the learning objectives	88.6 (148)	4.8 (8)	6.6 (11)
14b	The Three Rs (replacement, reduction and refinement) have been appropriately applied in the proposed animal use	87.4 (146)	5.4 (9)	7.2 (12)
15	Which of the following elements require additional guidance from the Canadian Council on Animal Care for animal use in teaching and training:			
15a	The objective of pedagogical merit reviews	62.3 (104)	22.2 (37)	15.6 (26)
15b	Criteria to address during the pedagogical review process	69.5 (116)	17.4 (29)	13.2 (22)
15c	How to establish when animal use is pedagogically justified	68.9 (115)	14.4 (24)	16.8 (28)
15d	The types of animal use in teaching/training that require pedagogical merit review	67.1 (112)	15.0 (25)	18.0 (30)
16	As part of the course development the instructor should review the Three Rs (replacement, reduction and refinement) for their proposed animal use	89.2 (149)	4.2 (7)	6.6 (11)
17	For the pedagogical merit review of the use of animals in teaching and training there should be standardized forms:			
17a	For reporting the proposed animal use from instructors for pedagogical merit review	70.1 (117)	18.0 (30)	12.0 (20)
17b	For reporting the results of the pedagogical merit review to the Animal Care Committee	65.3 (109)	24.0 (40)	10.8 (18)
18	The ethics of the proposed use of animals in teaching and training should be assessed during:			
18a	The pedagogical merit review	61.7 (103)	16.2 (27)	22.2 (37)
18b	The Animal Care Committee review	83.8 (140)	4.8 (8)	11.4 (19)
19	Students should be informed that the course has undergone a pedagogical merit review of the use of animals in teaching and training	81.4 (136)	13.8 (23)	4.8 (8)
20	The pedagogical merit review of the use of animals in teaching and training should evaluate if the use of animals is necessary for meeting the objectives of the learning session	88.6 (148)	4.8 (8)	6.6 (11)

n = number of participants

Participants were asked whether pedagogical merit review at their institution was effective. Many participants (59%) agreed that their pedagogical merit review process was effective, while some were in disagreement (13%) and several were (28%) neutral (Fig 2). Participants who had experience either as instructors in animal based teaching/training or conducting pedagogical merit review did not differ in their responses from those without these experiences; χ² (2, N = 167) = 1.61, *p* = 0.45; χ² (2, N = 167) = 4.09, *p* = 0.13.

One-hundred and one participants responded to question 21: what is the most significant challenge at your institution in the pedagogical merit review of the use of animals in teaching and training? Few participants had no challenge (6%) or were neutral in their response (8%), but most indicated that there was some challenge (86%). Participants who had no challenges either were unaware of any or had a system that worked well: “*None the process has been revamped recently and is working fine*”, and “*No major challenge of which I am aware*”. For those participants that indicated there were some challenges the most common emergent themes (> 10 comments) were burdensome, lack of expertise, lack of resources, and a lack of guidance from the CCAC. **Example statement for burdensome:** “*More paper work for instructors*. *The bulk of the work would likely be downloaded onto instructors*”, and “*Even relatively benign activities such as handling domestic animals or observing animal behaviour in the lab or in the wild (e*.*g*. *observing goldfish in aquaria) require such extensive documentation and justification that many instructors simply do not bother any longer to try to incorporate real animals into the courses”*. **Example statements for lack of expertise:** “*Finding a pool of experts outside our institution willing to provide a pedagogical merit review*”, and “*Appropriate individuals for evaluation; evaluating merit vs academic freedom*”. **Example statements for lack of resources**: “*The time required by a committee to evaluate all of the projects*”, and “*the limited number of researchers (professor/senior scholar)*”. **Example statements for lack of guidance:** “*lack of clear guidelines”*, *and “Lack of specific guidance from CCAC*”.

### Current CCAC Guidance/Policy (Questions 12, 14, 16, 18)

We assessed participant views on current CCAC policy for pedagogical merit review of animal studies for alternatives and Three Rs (question 14). Most participants agreed that both the Three Rs and the use of non-animal alternatives should be evaluated in a pedagogical merit review (89% and 87%). Most participants also agreed that the Three Rs should be assessed during the course development (question 16; Agree 89%, Disagree 7%, Neutral 4%). We asked participants whether ethics should be assessed during the ACC review or during the pedagogical merit review (question 18). Although many participants agreed that ethics should be reviewed during either the pedagogical merit review or ACC review; more (83% vs. 62%) agreed that the ACC should review ethics (Fig 3). We asked participants if institutions should treat the pedagogical merit review of animal-based teaching/training the same as scientific merit review for animal-based research (question 12) [6]. Many participants agreed (64.7%) but some disagreed (21.6%) or were neutral (13.8%; Fig 4).

### Proposed Changes to Peer Review Policy (Questions 9–11, 13, 15, 17, 19, 20)

#### Committee structure

A similar number of participants agreed and disagreed that it would be feasible to establish a single committee to address the pedagogical merit of animal-based teaching/training at their institution (38.3% and 36.5% respectively; Fig 5) and some were neutral about the feasibility of such a committee (25.1%; question 10; Fig 5). Participants who had experience with animal-based teaching/training as an instructor did not differ in their responses from those without experience (χ² (2, N = 167) = 5.47, *p* = 0.07), and participants who had experience reviewing the pedagogical merit of animal-based teaching/training also did not differ in their responses from those without experience (χ² (2, N = 167) = 1.30, *p* = 0.52). We asked participants to rate four separate committee structures for pedagogical merit review (question 9). Similar numbers of participants agreed and disagreed that pedagogical merit review should be conducted by a committee separate from the ACC, a dedicated pedagogical merit committee, or a curriculum committee(s) (Agree 45.5%, 43.7%, 42.5%; Disagree 35.9%, 35.3%, 34.1%; S1 Fig). Few participants agreed with the establishment of a national peer review committee (16.8%) and many disagreed (64.7%; S1 Fig); however, some participants indicated that external access to reviewers with relevant expertise would be helpful for small institutions, “… we are a small institution and are required to go outside of our institution… *A pool of experts should be available through the CCAC*”.

#### Committee membership

We asked participants who should be included on a committee that conducted pedagogical merit review (question 11). Most participants agreed that experts in education; instructors involved in animal-based teaching and training, and veterinarians should be on the committee (92.2%, 76.6%, 83.8%; S2 Fig). Many also agreed that community members, undergraduate students, and graduate students should also be members (58.1%; 52.1%; 61.1%; S2 Fig). Almost 10% more agreed that graduate students should be on the committee compared to undergraduate students. The greatest level of disagreement was with the involvement of community members (25.1%), undergraduate students (23.4%), and graduate students (12.0%). The difference between undergraduate and graduate student agreement/disagreement levels is likely because participants viewed graduate students as having more relevant experience as indicated in the comments (S2 Fig). Participants’ comments focused on appropriate expertise as criteria for involvement on the committee but differed in what this knowledge was. For instance, *“…need to be familiar with the type of animal research being conducted—wildlife work is very different than laboratory animal research*” and “*representatives who in one sense are 'community members' but beyond that can contribute very distinct and pertinent perspectives relating to animals particular to the region*. *For example*, *including an Aboriginal Elder who will speak to longstanding cultural and traditional values as it respects animals*, *including traditional teachings about animals*”. Participants’ comments about student involvement were either mixed “*If it is addressed by a separate committee*, *all of the above should be included*. *If it is done by curriculum committee*, *I don't see why a community rep*, *grads and undergrads could fit in such committee*”, and “*Undergraduate students may lack the required maturity and knowledge”*. Some participants also indicated other members that should be considered: aboriginal, agronomists, animal care technicians and animal welfare experts”.

#### Review content

We asked participants what pedagogical merit review should evaluate on four sub-items (question 13). Most participants agreed that the review should evaluate *if the use of animals is essential for meeting the education objectives of the*: learning session (92.8%); the course (94.6%); the program (88.0%) and many agreed that the review should be for the entire course (77.8%; S3 Fig). We also asked participants if the review should evaluate whether the animal use is *necessary* for the learning session (question 20) in contrast with the term *essential* used in question 13. Most participants agreed (essential 88.6% vs. necessary 92.8%) regardless of the term used, and few switched indicating strong reliability of the item. We also asked participants which elements required further guidance from the CCAC on four sub-items: the justification, criteria, types of animal use, and objectives (question 15). Many participants agreed that additional guidance from the CCAC is required for each of the four items: 1) how to establish pedagogical merit, (69.5%); 2) what criteria to review (68.9%); 3) what type of animal use require a pedagogical merit review (67.1%); 4) the objective of the review (62.3%; S4 Fig). Approximately an equal number of participants responded either neutrally or disagreed (Neutral, 14.4%–22.2%; Disagree, 13.2%–18.0%; S4 Fig).

#### Communication of review

Many participants agreed with the used of standardized forms for reporting proposed animal-based teaching/training by instructors (65.3%) or for submission of a pedagogical merit review to the ACC (70.1%; question 17). Although there was no comment box for this question a few participants commented in the space provided on the subsequent question and indicated that they disagreed with the use of a standardized form only if this would be imposed nationally as opposed to the use of a standardized form developed by the local institution. Most participants agreed that students should be informed that the animal-based course they are taking has undergone a pedagogical merit review (81.4%; question 19).

### Aspect of Evaluation and Emergent Themes

**Fig 6:**
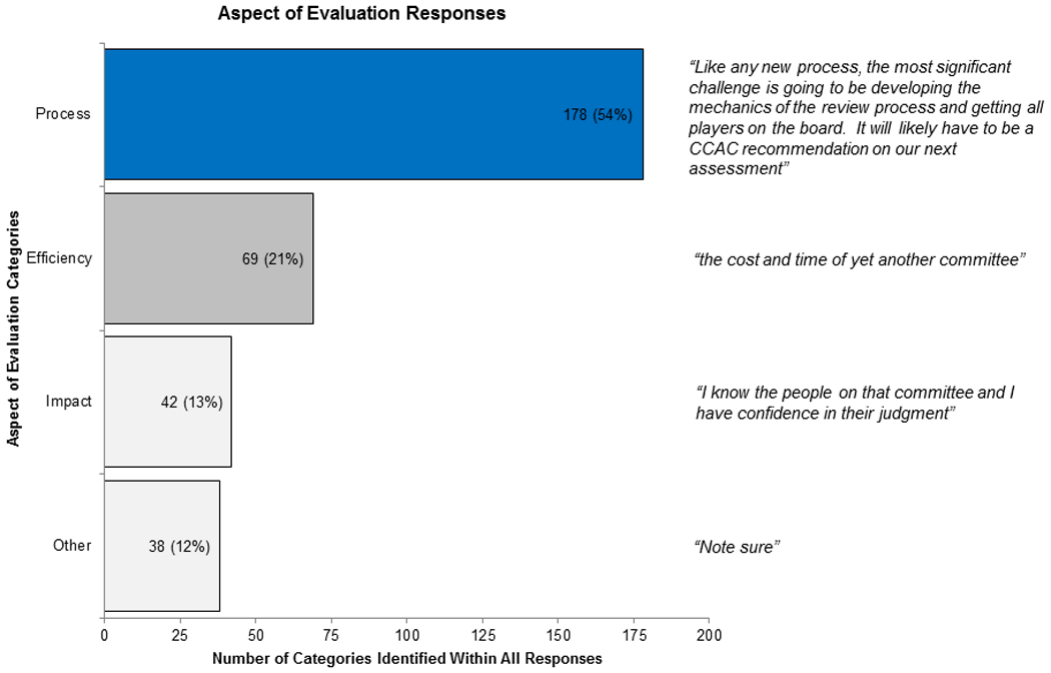
Aspect of Evaluation Responses.

A total of 324 narrative comments (median 27; range: 12–101 per question) were added in the optional comment boxes. We excluded comments to question 11 (25 comments) because they were recommendations for committee membership. We reviewed the remaining 299 comments for each of the four aspects of evaluation (*impact*, *efficiency*, *process*, *other*). Comments about *process* accounted for half (54%) of all responses with *efficiency* accounting for slightly less than a quarter (21%), and *impact* accounting for the least of the three (13%; Fig 6). Representative comments are included in the Figures.

**Fig 7:**
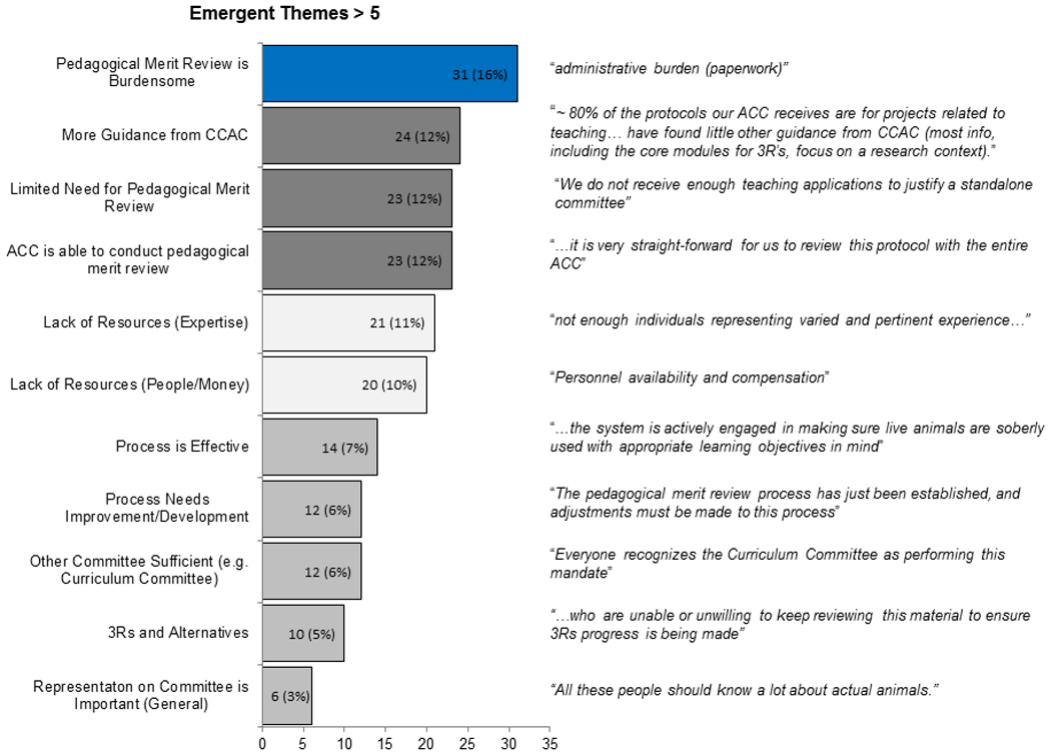
Emergent Themes > 5.

For emergent themes we reviewed the same 299 comments and we identified ten themes that each had greater than five comments (from 196 of the 299 comments; Fig 7). The most common theme was that *pedagogical merit review was burdensome* (16%); which when combined with *a limited need for the review* (12%), *a lack of resources* (expertise; 11%), and *a lack of resource*s (people/money; 10%) accounted for 49% of the comments (Fig 7). Participants’ comments also indicated that *more guidance from the CCAC* (12%) was a priority, and confidence that the *ACC is able to conduct pedagogical merit review* (12%; Fig 7). As above, representative comments are included in the Figures.

### Participant Demographics

Two-hundred and sixty-six individuals responded by starting the survey. One-hundred and sixty-seven completed the survey in full (63% completion rate). The majority of participants were from academic institutions (71%), although several participants were from government institutions (22%; Table 2). Participants were drawn from all provinces except Newfoundland and Labrador (Table 3). Participants self-identified their current occupation and were given the option of selecting multiple occupations. Most participants self-identified at least one of their occupations as administrative (27%), instructor (23%), research scientist (23%), or veterinarian (17%; Table 2). Only a few participants identified themselves as students (3%), technicians (6%) or “other” (2%; ethicist, lay member etc.). Many participants indicated that they have been instructors for animal-based teaching/training (69%), which was higher than the number that identified their occupation as being an instructor (Table 3). This result suggests that many participants worked as instructors in the past but currently do not self-identify with that role. Most participants reported having been involved in animal-based courses as students (85%), and over half of the participants (60%) had experience reviewing the pedagogical merit of animals for teaching/training (Table 3).

**Table 2:** Participant Characteristics (N = 167).

Attribute	Responses: n (%)								
Date of Birth	1930–1939	1940–1941	1950–1959	1960–1969	1970–1979	1980–1989	1990–1999	N.R.	
	1 (0.6%)	12 (7.2%)	43 (25.7%)	56 (33.5%)	33 (19.8%)	13 (7.8%)	2 (1.2%)	7 (4.2%)	
Gender	Female	Male	N.R.						
	80 (47.9%)	81 (48.5%)	6 (3.6%)						
Institution	Academic	Government	Industry	Non-Profit	Other				
	119 (71.3%)	36 (21.6%)	4 (2.4%)	4 (2.4%)	4 (2.4%)				
Province	AB	BC	MB	NB	NS	ON	PEI	QC	SK
	14 (8.4%)	21 (12.6%)	19 (11.4%)	5 (3.0%)	10 (6.0%)	34 (20.4%)	13 (7.8%)	35 (21.0%)	16 (9.6%)
Position*	Admin	Instructor	Scientist	Undergrad	Grad Student	Veterinarian	Other		
	53 (21.8%)	53 (21.8%)	55 (22.6%)	2 (0.8%)	5 (2.1%)	41 (16.9%)	34 (14.0%)		

*Participants could select more than one position (N = 243 responses)

N.R. = No Response

Provinces: AB Alberta, BC British Columbia, MB Manitoba, NB New Brunswick, NS Nova Scotia, ON Ontario, PEI Prince Edward Island, QC Québec, SK Saskatchewan.

**Table 3:** Participant Experience (N = 167).

Animal Use	No, n (%)	Yes, n (%)
As Instructor	51 (30.5%)	116 (69.5%)
As Student	25 (15.0%)	142 (85.0%)
Pedagogical Merit Review	66 (39.5%)	101 (60.5%)

## Discussion

The feedback from participants identified clear views about how the peer review of pedagogical merit may be improved. Participants were focused on the process of peer review and the burden/resources required for its completion. The review itself, as well as the need for a separate committee place additional burdens on already limited resources and expertise at institutions, making implementation difficult. This sense of burden aligns with similar findings from the National Science Foundation’s report on administrative workload [20] in the United States and a survey of Canadian animal-based researchers’ views on the Three Rs [21].

### Current Practice at Institutions

Although many participants indicated that their pedagogical merit review process was effective, almost a third were neutral. Most of these participants indicated that they faced some challenges, with burden, and lack of expertise or resources cited frequently. A lack of resources and the perceived additional burden of the review are likely related. Similarly, the perceived effectiveness of the review may also be related to the available resources (i.e. expertise). Although beyond the scope of our survey questions, identifying factors that result in ACCs perceiving themselves as effective should be explored in future research. A lack of expert reviewers is a familiar theme for peer review of research and grants as well. To address this deficit, the CCAC could consider exploring alternative approaches, such as facilitating a college of expert reviews that could be utilized by local ACCs where local expertise is lacking.

### CCAC Guidance/Policy—Three Rs

There was strong support for the Three Rs and the use of alternatives as well as ethics review; although there was more support for reviewing ethics during the ACC review of a protocol, rather than during the pedagogical merit review. The overall increase in the number of animals in teaching/training as enumerated by the annual CCAC animal data report is contrary to the responses of participants that indicated there was limited need for this type of review. In particular some participants elaborated that the limited need was a result of reducing/replacing animal-based teaching/training. This schism between the CCAC annual animal data report and the perception of participants may contribute to the sense of unnecessary administrative burden by individuals who see reduction and replacement happening at an individual or institutional level while national data indicates the opposite trend. The CCAC animal data does not provide metrics for institutional, individual, or protocol level changes which may mask subtleties in animal use. For instance, instructors may be reducing the numbers of animals per protocol or replacing animals completely, but this will not be detected due to the level of detail in the collected animal data. Improved sensitivity of reporting would be needed to detect these subtleties, which might better inform policy, reduce frustration from instructors, and better inform the public about efforts by individuals and institutions to reduce and replace animal-based teaching/training.

### Committee Structure & Membership

Some participants were either at institutions or on committees that had high volumes of teaching/training protocols (e.g. “~80% of the protocols our ACC receives are for projects related to teaching…”) whereas others clearly indicated that there was limited need for this type of review making a stand-alone committee an unnecessary use of resources. Participants also differed on the level of external support they desired with some responding more positively to CCAC guidance and standardization while others indicated that standardization should be left up to institutions. These dichotomies in the responses are likely a result of the variety of institutions that participate in the CCAC program which includes large research universities, small teaching colleges, government departments, and pharmaceutical companies. Successful policy changes will require balancing standardization and flexibility in peer review guidance to meet the needs of all of these groups. For instance, peer review at institutions with large numbers of protocols for teaching/training may be best served by a single dedicated committee for pedagogical merit of animal-based teaching/training use whereas institutions that receive few may be best served by having a sub-committee of the ACC or curriculum committee conduct the review. Policy should reflect this. Small institutions may also benefit from access to a pool of qualified peer reviewers and standardized forms etc. curated at a national level. Smaller institutions could contact these external reviewers to assist in the pedagogical merit review similar to the manner in which peer review of publications relies on external reviewers for expertise.

### Review Content & Communication

Participants mostly agreed that pedagogical merit of animal-based teaching/training should include a review of the education objectives of the learning session, course, and program. Reviewing the pedagogical merit of the course itself (i.e. separately from the animal involvement) was viewed less favourably by participants. This indicates that participants generally agreed that the scope of the merit review could encompass everything from the particular learning session to the entire program but only as it relates to the involvement of the animal in teaching/training. Opposing views were readily apparent in the comments such as those of a participant who preferred the term *necessary* over *essential* and another who felt that *essential* was too restrictive. Participants also generally agreed that further guidance from the CCAC was required on how to establish merit, the criteria, type of animal use, and objective of the review. However, participants did not agree to these items as strongly and the comments indicated concern that the guidance should allow flexibility. Similarly, participants were generally in favour of standardized forms although a few participants indicated that these should be standardized at the local level and not nationally. Communicating to students that the course has undergone a specific review for the pedagogy of animal use was strongly supported and may increase awareness of oversight system for animal use.

### Conclusion

This survey of the peer review of pedagogical merit for animal use is to our knowledge the first ever completed. Although many participants agreed that their pedagogical merit review is effective, we did not directly assess effectiveness by evaluating the protocols themselves. Future studies should directly assess the effectiveness of the peer review of animal-based protocols as has been done for peer review of journal articles and grants [22,23]. Course evaluations also provide another means of assessing the pedagogical value of the animal use from students’ perspective post implementation of the course. Clearly the administrative burden of additional committees and standards must be taken into account for any changes in the policy or guidelines that are implemented by policy makers. Different institutions have varying levels of need for this type of review and resources available to conduct them. Policy will require a balance between standardization and flexibility to ensure that they are applicable to the local context where it is implemented. In addition, it may be useful for policy makers to re-evaluate the metrics for how the progress in the Three Rs is assessed at the national level to capture local improvements that may be masked by national trends.

## Supporting Information

S1 Appendix Survey. (PDF)

S1 Fig The pedagogical merit review of the use of animals in teaching and training should be conducted by. (TIF)

S2 Fig A pedagogical merit review of the use of animals in teaching and training should be conducted by a group that includes. (TIF)

S3 Fig The pedagogical merit review of the use of animals in teaching and training should evaluate. (TIF)

S4 Fig Which of the following elements require additional guidance from the Canadian Council on Animal Care for animal use in teaching and training. (TIF)
