# Supplementary material for: Pedagogical Merit Review of Animal Use for Education in Canada
Source: PLoS One. 2016 Jun 28;11(6):e0158002. doi: 10.1371/journal.pone.0158002 (PMC4924868; doi:10.1371/journal.pone.0158002)
Supplement: S1 Appendix — (PDF) [file pone.0158002.s001.pdf]

<b>Pedagogical Merit Review of the Use of Animals in Teaching and

Letter of Information

You are invited to participate in an opinion survey investigating the pedagogical merit review of the use of animals in teaching and training being conducted by Dr. Marc Avey and Dr. Gilly Griffin of the Canadian Council on Animal Care.

For the purpose of this survey, pedagogical merit refers to a distinction given to a process, material, or other concept that demonstrates and validates learning by the student. The purpose of this research is to solicit feedback on the current practices and guidance on pedagogical merit review of the use of animals in teaching and training. This information will then be used to inform future revisions to policies and guidance documents relating to pedagogical merit review processes.

We greatly appreciate your answers; however, you should not feel obliged to answer any material that you find objectionable or that makes you feel uncomfortable. You may withdraw at any time without consequence. If you withdraw prior to completing and submitting the survey, all data entered will be permanently removed.

Your identity will not be recorded and therefore your anonymity will be protected. To help us ensure anonymity, please do not put your name on any response. The results of this research may be published in professional journals or presented at scientific conferences, but any such presentations will report only aggregated findings. This may include the use of short, anonymous quotes selected so as not to breach individual confidentiality.

Should you be interested in receiving a copy of the study findings or a link to the website highlighting these findings, you can contact Dr. Griffin at ggriffin@ccac.ca (613) 238-4031 ext 225. Any questions about study participation may be directed to Dr. Avey at mavey@ccac.ca (613) 238-4031 ext 249. Any ethical concerns about this study may be directed to the Institutional Review Board Services at info@irbservices.com (905) 727-7989.

This study has been approved by the Institutional Review Board Services according to the recommended principles of Canadian ethics guidelines. Thank you for your interest in participating in this study.

Consent Decision

The first question of the survey will ask for your consent for our team to analyze and disseminate results based on your answers. If you select “yes” you will have agreed to participation in our study, if you select “no” you will have declined to participate in the this survey.

***1. By selecting "I agree to participate" below you confirm that you:**

A) Understand what is required based on reading the letter of information

B) Understand that your participation is voluntary and you are free to withdraw at any time

C) Understand the provisions for confidentiality

I agree to participate

◯ Yes ◯ No

Demographics

First we will ask you a few demographics questions then we will proceed with the opinion survey.

2. Please indicate your age and gender

	Year Born	Gender
Age and Gender		

*3. Please indicate your primary institution type and province/territory

	Primary Institution Type	Province/Territory
Institution and Province/Territory		

4. Current primary institution & faculty, department, program etc. (optional)

*5. Current occupation(s): Please select all that apply

- Administrator
- Instructor
- Research Scientist
- Student (Undergraduate)
- Student (Graduate)
- Veterinarian
- Other (please specify below)

Please Specify

*6. Have you ever used animals in teaching and training as:

	Yes	No
an instructor		
a student		

*7. Have you ever reviewed the pedagogical merit of the use of animals in teaching and training?

- Yes
- No

Opinion Survey

Please rate the extent to which you agree or disagree with the following statements. Comments are optional.

*8. At your institution, the current pedagogical merit review process of the use of animals in teaching and training is effective.

- ◯ Strongly Disagree
- ◯ Disagree
- ◯ Neutral
- ◯ Agree
- ◯ Strongly Agree

Comment

*9. The pedagogical merit review of the use of animals in teaching and training should be conducted by:

	Strongly Disagree	Disagree	Neutral	Agree	Strongly Agree
A committee(s) that is separate from the Animal Care Committee	◯	◯	◯	◯	◯
A curriculum committee(s)	◯	◯	◯	◯	◯
A single committee dedicated to the pedagogical merit review of the use of animals	◯	◯	◯	◯	◯
A national peer review committee	◯	◯	◯	◯	◯

Other (please specify)

***10. At your institution, it is feasible to establish a single committee specifically to address the pedagogical merit of the use of animals in teaching and training**

- Strongly Disagree
- Disagree
- Neutral
- Agree
- Strongly Agree

Comment

***11. A pedagogical merit review of the use of animals in teaching and training should be conducted by a group that includes:**

	Strongly Disagree	Disagree	Neutral	Agree	Strongly Agree
Community members					
Experts in education					
Instructors who use animals in teaching/training					
Students (Undergraduate)					
Students (Graduate)					
Veterinarians					

Other (please specify)

***12. Institutions should treat the pedagogical merit reviews of the use of animals in teaching and training in the same manner as scientific merit reviews**

- Strongly Disagree
- Disagree
- Neutral
- Agree
- Strongly Agree

Comment

***13. The pedagogical merit review of the use of animals in teaching and training should evaluate:**

	Strongly Disagree	Disagree	Neutral	Agree	Strongly Agree
The overall pedagogical merit of the course that the animal use takes place in	○	○	○	○	○
If the use of animals is essential for meeting the education objectives of the learning session	○	○	○	○	○
If the use of animals is essential for meeting the education objectives of the course	○	○	○	○	○
If the use of animals is essential for meeting the education objectives of the program	○	○	○	○	○

Comment

***14. The pedagogical merit of the use of animals in teaching and training should determine whether:**

	Strongly Disagree	Disagree	Neutral	Agree	Strongly Agree
The use of non-animal alternatives could meet the learning objectives	○	○	○	○	○
The Three Rs (replacement, reduction and refinement) have been appropriately applied in the proposed animal use	○	○	○	○	○

***15. Which of the following elements require additional guidance from the Canadian Council on Animal Care for animal use in teaching and training:**

	Strongly Disagree	Disagree	Neutral	Agree	Strongly Agree
The objective of pedagogical merit reviews	◯	◯	◯	◯	◯
Criteria to address during the pedagogical review process	◯	◯	◯	◯	◯
How to establish when animal use is pedagogically justified	◯	◯	◯	◯	◯
The types of animal use in teaching/training that require pedagogical merit review	◯	◯	◯	◯	◯

Other (please specify)

***16. As part of the course development the instructor should review the Three Rs (replacement, reduction and refinement) for their proposed animal use**

- ◯ Strongly Disagree
- ◯ Disagree
- ◯ Neutral
- ◯ Agree
- ◯ Strongly Agree

***17. For the pedagogical merit review of the use of animals in teaching and training there should be standardized forms:**

	Strongly Disagree	Disagree	Neutral	Agree	Strongly Agree
For reporting the proposed animal use from instructors for pedagogical merit review	◯	◯	◯	◯	◯
For reporting the results of the pedagogical merit review to the Animal Care Committee	◯	◯	◯	◯	◯

***18. The ethics of the proposed use of animals in teaching and training should be assessed during:**

	Strongly Disagree	Disagree	Neutral	Agree	Strongly Agree
The pedagogical merit review	◯	◯	◯	◯	◯
The Animal Care Committee review	◯	◯	◯	◯	◯

Other (please specify)

***19. Students should be informed that the course has undergone a pedagogical merit review of the use of animals in teaching and training**

- ◯ Strongly Disagree
- ◯ Disagree
- ◯ Neutral
- ◯ Agree
- ◯ Strongly Agree

<b>Pedagogical Merit Review of the Use of Animals in Teaching and

***20. The pedagogical merit review of the use of animals in teaching and training should evaluate if the use of animals is necessary for meeting the objectives of the learning session**

- ◯ Strongly Disagree
- ◯ Disagree
- ◯ Neutral
- ◯ Agree
- ◯ Strongly Agree

21. What is the most significant challenge at your institution in the pedagogical merit review of the use of animals in teaching and training?

22. Any other comments or feedback?

Please click "Done" to submit your survey.

Thank-you for participating.

Should you be interested in receiving a copy of the study findings or a link to the website highlighting these findings, you can contact Dr. Griffin at ggriffin@ccac.ca (613) 238-4031 ext 225. Any questions about study participation may be directed to Dr. Avey at mavey@ccac.ca (613) 238-4031 ext 249. Any ethical concerns about this study may be directed to the Institutional Review Board Services at info@irbservices.com (905) 727-7989.
