# Supplementary figures and images for: Pedagogical Merit Review of Animal Use for Education in Canada
Source: PLoS One. 2016 Jun 28;11(6):e0158002. doi: 10.1371/journal.pone.0158002 (PMC4924868; doi:10.1371/journal.pone.0158002)

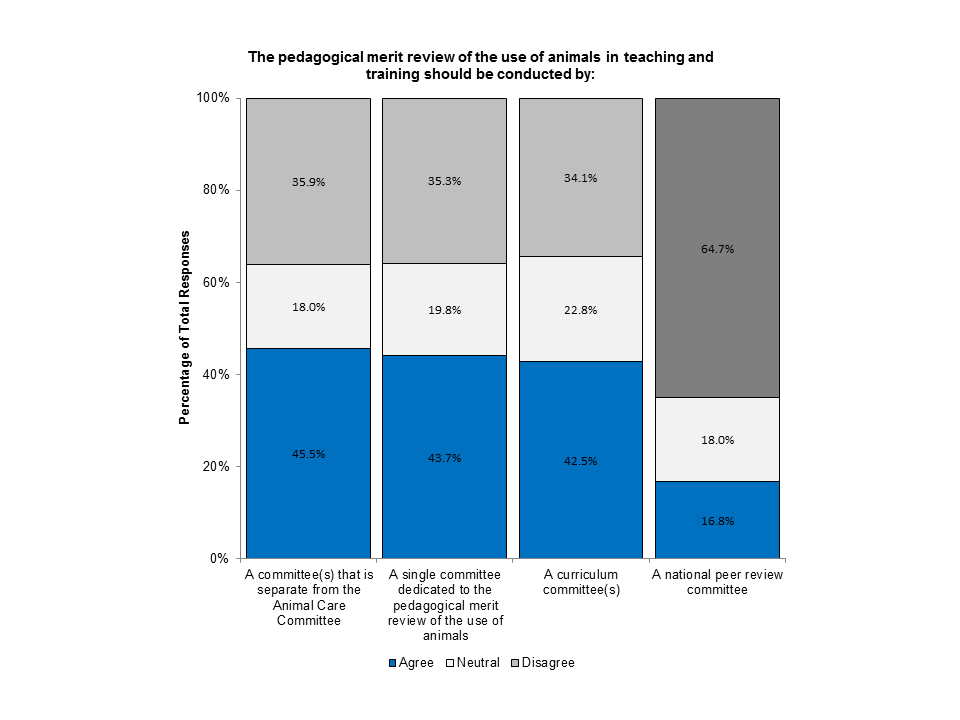

Supplement: S1 Fig — (TIF) [file pone.0158002.s002.tif]

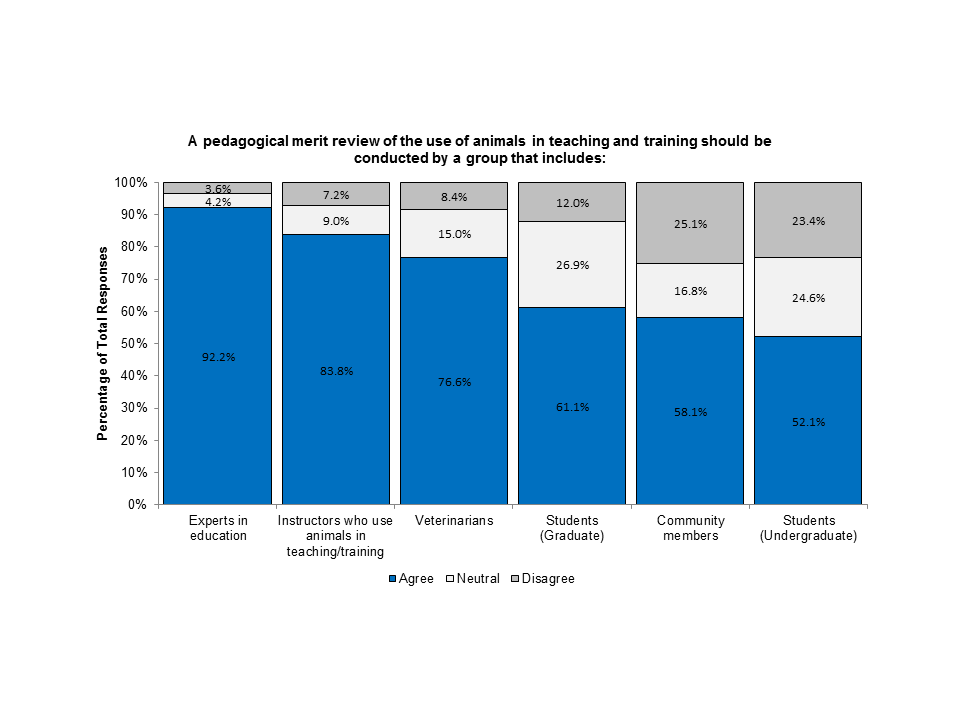

Supplement: S2 Fig — (TIF) [file pone.0158002.s003.tif]

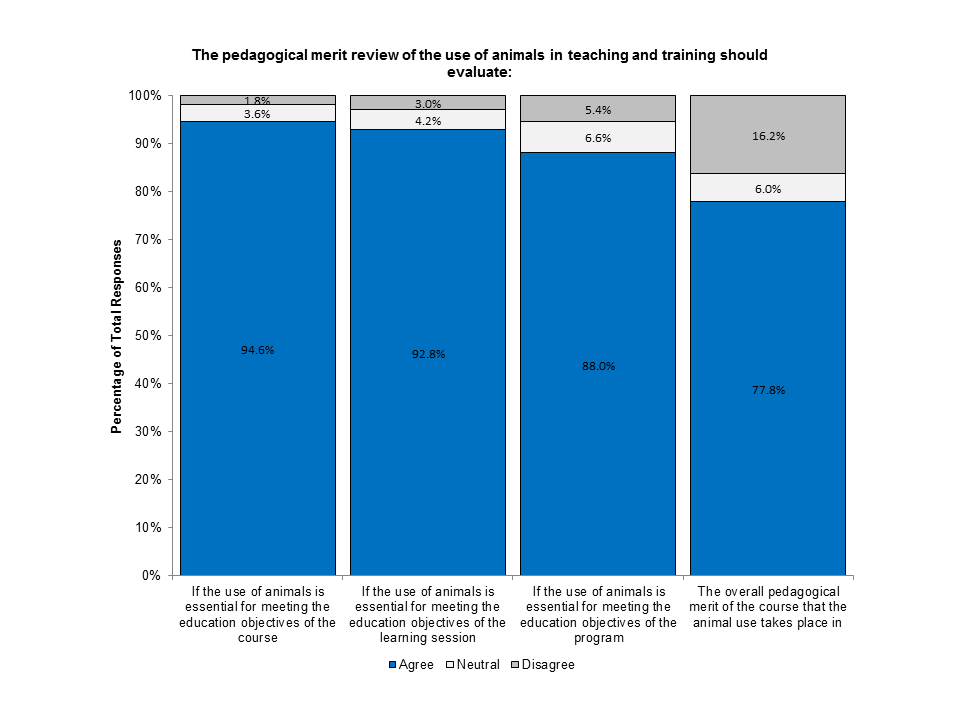

Supplement: S3 Fig — (TIF) [file pone.0158002.s004.tif]

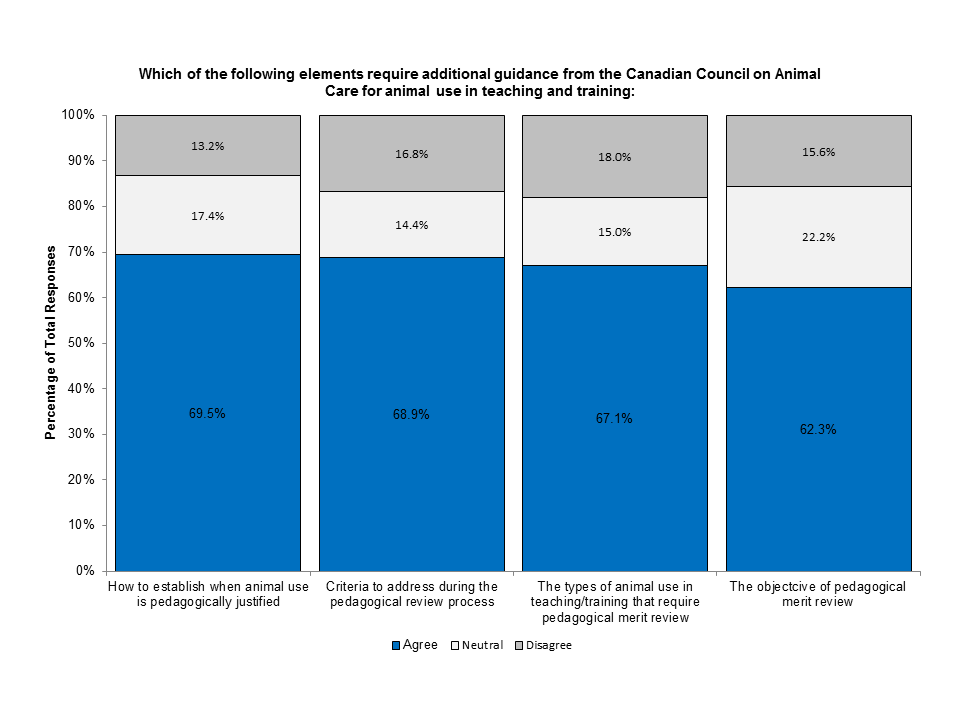

Supplement: S4 Fig — (TIF) [file pone.0158002.s005.tif]
